# Radical Cure of External Pulmonary Hernia

**Published:** 1885-04

**Authors:** 


### III.—RADICAL CURE OF EXTERNAL PULMONARY HERNIA.

Guglielmi reports a case of a cow being hooked in the thorax so as to cause a large hole, and the exudation of a piece of the lung weighing half a pound. It was dark-blue in color and atalectatic. A strong ligature was placed around the mass, the wound being daily dressed with carbolic solution. In the course of ten days the piece of lung sloughed off, the external wound in the thoracic wall healing nicely. Animal remained completely well.—*Clinica Veterinaria.*

